# Antimicrobial Effects of *Inula viscosa* Extract on the In Situ Initial Oral Biofilm

**DOI:** 10.3390/nu13114029

**Published:** 2021-11-11

**Authors:** Hannah Kurz, Lamprini Karygianni, Aikaterini Argyropoulou, Elmar Hellwig, Alexios Leandros Skaltsounis, Annette Wittmer, Kirstin Vach, Ali Al-Ahmad

**Affiliations:** 1 Department of Operative Dentistry and Periodontology, Faculty of Medicine and Medical Center, University of Freiburg, 79085 Freiburg, Germany; hannahkurz@gmx.net (H.K.); elmar.hellwig@uniklinik-freiburg.de (E.H.); 2 Clinic of Conservative and Preventive Dentistry, Center of Dental Medicine, University of Zurich, 8006 Zurich, Switzerland; Lamprini.Karygianni@zzm.uzh.ch; 3 Department of Pharmacognosy and Natural Products Chemistry, Faculty of Pharmacy, National and Kapodistrian University of Athens, 157 72 Athens, Greece; katarg@pharm.uoa.gr (A.A.); skaltsounis@pharm.uoa.gr (A.L.S.); 4 Institute of Medical Microbiology and Hygiene, Faculty of Medicine, University of Freiburg, 79085 Freiburg, Germany; annette.wittmer@uniklinik-freiburg.de; 5 Institute for Medical Biometry and Statistics, Faculty of Medicine and Medical Center, University of Freiburg, 79085 Freiburg, Germany; kv@imbi.uni-freiburg.de

**Keywords:** *Inula viscosa*, initial adhesion, colony-forming units (CFU), live/dead staining, fluorescence microscopy

## Abstract

Given the undesirable side effects of commercially used mouth rinses that include chemically synthesized antimicrobial compounds such as chlorhexidine, it is essential to discover novel antimicrobial substances based on plant extracts. The aim of this study was to examine the antimicrobial effect of *Inula viscosa* extract on the initial microbial adhesion in the oral cavity. Individual test splints were manufactured for the participants, on which disinfected bovine enamel samples were attached. After the initial microbial adhesion, the biofilm-covered oral samples were removed and treated with different concentrations (10, 20, and 30 mg/mL) of an *I. viscosa* extract for 10 min. Positive and negative controls were also sampled. Regarding the microbiological parameters, the colony-forming units (CFU) and vitality testing (live/dead staining) were examined in combination with fluorescence microscopy. An *I. viscosa* extract with a concentration of 30 mg/mL killed the bacteria of the initial adhesion at a rate of 99.99% (log_10_ CFU value of 1.837 ± 1.54). Compared to the negative control, no killing effects were determined after treatment with *I. viscosa* extract at concentrations of 10 mg/mL (log_10_ CFU value 3.776 ± 0.831; median 3.776) and 20 mg/mL (log_10_ CFU value 3.725 ± 0.300; median 3.711). The live/dead staining revealed a significant reduction (*p* < 0.0001) of vital adherent bacteria after treatment with 10 mg/mL of *I. viscosa* extract. After treatment with an *I. viscosa* extract with a concentration of 30 mg/mL, no vital bacteria could be detected. For the first time, significant antimicrobial effects on the initial microbial adhesion in in situ oral biofilms were reported for an *I. viscosa* extract.

## 1. Introduction

Biofilms consist of microbial cells that are irreversibly attached to a surface or interface and embedded in a matrix of extracellular polymeric substances that are produced by the microorganisms themselves [1]. Furthermore, the microorganisms within a biofilm have altered growth characteristics and gene expression patterns as compared to their planktonic counterparts [1]. The oral cavity is an ideal niche for biofilm formation, which commences with the development of the acquired salivary pellicle (conditioning layer) on which the early colonizers, including oral streptococci, *Actinomyces* spp., and *Veillonella* spp., adhere in an initial phase [2]. After this initial phase, the different microbial species produce a diverse extracellular matrix and multiply to form a thick mature oral biofilm [3,4]. The oral biofilm consists of a plethora of different microbial species, including bacterial species of the genera *Streptococcus*, *Actinomyces*, *Fusobacterium*, *Rothia*, *Veillonella*, *Prevotella*, *Tannerella*, and *Porphyromonas*, *Neisseria,* and *Gemella* [5,6,7,8]. In addition to bacteria, fungi such as members of the Candida, protozoa, and Archaea species have been detected in oral biofilm [8]. As biofilms cause serious infections in different fields of medicine, novel and alternative methods such as the use of natural products are required to prevent biofilm formation [9]. The oral biofilm consists of more than 700 different bacterial species [10,11]. Biofilms are up to 1000 times more resistant to antibiotics compared to equivalent planktonic microorganisms that remain unbound in free suspension [12]. Alterations in the microbial composition in the oral cavity can lead to an increased risk of tooth decay, periodontitis, and periimplantitis [13]. Alternative disinfection methods based on plant extracts recently started receiving increased attention as potential substitutes for known antibacterial agents. Substantial efforts have been made to identify alternative substances to substitute commonly used mouth rinses such as chlorhexidine (CHX) [14]. Although CHX is still regarded as the gold standard in the prevention of plaque formation and the treatment of gingivitis, it causes diverse side effects including CHX-resistance in oral bacteria [15] and reversible taste disorders [16]. Additionally, CHX use results in undesirable effects such as a discoloration of the tongue, composite fillings, and teeth [17,18]. Moreover, due to the increasing number of antibiotic-resistant microorganisms, plant extracts are gaining importance as potent alternatives to circumvent resistance and remove biofilms [19]. Such plant extracts could feature similar antimicrobial and anti-inflammatory behavior to existing treatments but deliver remedial effects more gently, thereby reducing side effects [20]. In 2014, the World Health Organization (WHO) published recommendations in which they emphasized the importance of traditional and alternative phytomedicine for the well-being of mankind and presented a large number of proposals to establish more plant-based medicine and drugs [21]. Since nature yields a large, complex, and mostly unexplored reservoir of phytotherapeutic agents that provide alternatives to common pharmaceuticals for oral antibiosis, it is of crucial importance to find effective antimicrobial mouth rinses based on natural substances [14]. Phytotherapy is used in modern dentistry, mainly for the anti-inflammatory, antibiotic, analgesic, or sedative effects of herbal remedies or as a component of root canal irrigations [22].

To date, several studies have been conducted to examine the effects of natural extracts on different bacterial species in vitro, ex vivo, and in situ [9]. It could be shown that especially *Vitis vinifera*, *Pinus* spp., *Coffea canephora*, *Clonorchis sinensis*, *Vaccinium macrocarpon*, *Psidium cattleianum,* and Manuka honey have a significant antimicrobial effect on oral biofilms. The antibacterial, antiviral, and antifungal effects of the tested *Inula viscosa* extract have been confirmed in several studies [23,24,25]. Especially with regard to oral pathogens, the effectiveness of the ethyl acetate extract and the methanol extract of *I. viscosa* against both Gram-negative (*Porphyromonas gingivalis*, *Prevotella intermedia*, *Fusobacterium nucleatum*) and Gram-positive (*Staphylococcus aureus*, *Streptococcus mutans*, *Streptococcus sobrinus*, *Streptococcus oralis*) species, and also against the fungus *Candida albicans* in concentrations between 0.15 and 5.00 mg/mL, was shown [14].

The *I. viscosa* plant (*Dittricha viscosa*) belongs to the Asteraceae family and mainly grows in the Mediterranean area [26]. *I. viscosa* shrubs can be found in southern Europe, Turkey, and the Middle East. Due to its adaptive behavior, *I. viscosa* also grows as a neophyte in Great Britain, Belgium, and North America [27]. *I. viscosa* is a stem hemicryptophyte or nanophanerophyte that reaches heights of 40 to 130 cm. The leaves are 6 to 12 mm wide, slim to lancet-like, sticky, and have an unpleasant smell [28]. In a previous study, the effect of *I. viscosa* on selected oral bacterial species was tested in vitro and yielded minimum inhibitory concentrations (MIC) ranging from 0.07 mg/mL (*P*. *gingivalis*) up to 2.50 mg/mL (*S*. *sobrinus*), and showed the elimination of obligate anaerobes such as *P. gingivalis* at a minimal bactericidal concentration (MBC) of 0.15 mg/mL [14]. To date, there are no clinical data on the antimicrobial impact of *I*. *viscosa* on in situ initial oral biofilms. *I. viscosa* has been used for a long time as a herb in folk medicine to treat skin inflammations and diseases such as scabies [29,30]. Different health benefits were reported for the use of *I. viscosa* extract, including its anticancer, antioxidant, antifungal, antibacterial, and hypoglycemic effects [31]. Phytochemical analysis of *I. viscosa* revealed the presence of compounds that have the potential to be used as food additives such as flavonoids, triterpenoids, and sesquiterpenoids [31,32]. Additionally, the application of *I. viscosa* tea yielded a significant reduction of adherent bacteria of the initial in vivo oral biofilm without any negative impact on the acid protective properties of the salivary pellicle [27]. Subsequently, the aforementioned reports on *I. viscosa* show the great potential for this species to be used in maintaining oral health, especially due to the direct contact of its different ingredients with the oral mucosa. Therefore, the antimicrobial effect of *I*. *viscosa* on the initial adhesion of in situ oral biofilms was investigated in the present study to acquire new knowledge in dental phytotherapy. The aim of the present study was to evaluate if there are any antimicrobial effects of *I. viscosa* extract on the initial oral biofilm and hence to clarify if there is a potential for using this extract for the treatment of dental diseases caused by the oral biofilm.

## 2. Materials and Methods

All reagents used in the study are depicted in Table S1 (Supplementary Material).

### 2.1. Selection of Study Participants and Test Specimens

Six healthy volunteers participated in this study and wore appliances that included bovine enamel samples to acquire in situ initial oral biofilm samples. The volunteers were between 23 and 50 years old and had neutral saliva (pH 6.6–7.4) with an average salivary flow rate of 1.49 mL/min. None of the volunteers suffered from carious lesions, insufficient restorations, or periodontal diseases at the time of wearing the specimen. A stable hold of the support rails and sufficient space for the enamel platelets was ensured. The prerequisites for participation in the study included: (i) no use of antibiotics and mouthwashes in the three months prior to wearing the appliance, (ii) no pregnancy or breastfeeding, (iii) no systemic diseases, and in addition, (iv) no oral hygiene was to be carried out in the two hours prior to wearing the appliance, (v) the consumption of food, liquids, alcohol, and nicotine was not permitted while wearing the appliances, and finally, (vi) the subjects had not participated in any other clinical examination up to 30 days before the commencement of the study. The use of chewing gum was also not permitted while the splint was worn. All participants were non-smokers. Since all volunteers remained in the Department for Operative Dentistry and Periodontology while wearing the splint systems, all prerequisites for the sample collection were ensured. The test persons provided written consent prior to participation and the study was approved by the Ethics Committee of the University of Freiburg (no. 91/31).

Bovine front teeth were extracted from caries- and bovine spongiform encephalopathy (BSE)-free cattle. The test specimens were manufactured as described earlier in detail [33] so that the resulting enamel platelets had a constant thickness of 1.5 mm, which corresponded to a total surface area of 19.63 mm^2^. The enamel side of the cylinder was ground and polished flat on a hand sanding pad with sandpaper in ascending order of grain size (220–4000 grit), so that no irregularities or facets could be seen under a light microscope (Wild M3Z; Leica GmbH, Wetzlar, Germany) prior to being cleansed.

The disinfection of the bovine enamel platelets took place in different solutions in an ultrasonic bath. First, the produced platelets were exposed to a 3% sodium hypochlorite (NaOCl) solution and ultrasound in plastic cups for three minutes to remove the superficial smear layer. After air drying, the next step was the disinfection with 70% ethanol in an ultrasonic bath for a further three minutes. Finally, the platelets were treated twice for 10 min each in double-distilled water in an ultrasonic bath. Following the disinfection protocol, the enamel samples were stored in distilled water (H_2_O) for at least 24 h in order to ensure the formation of a hydration layer [34].

**Figure 1:**
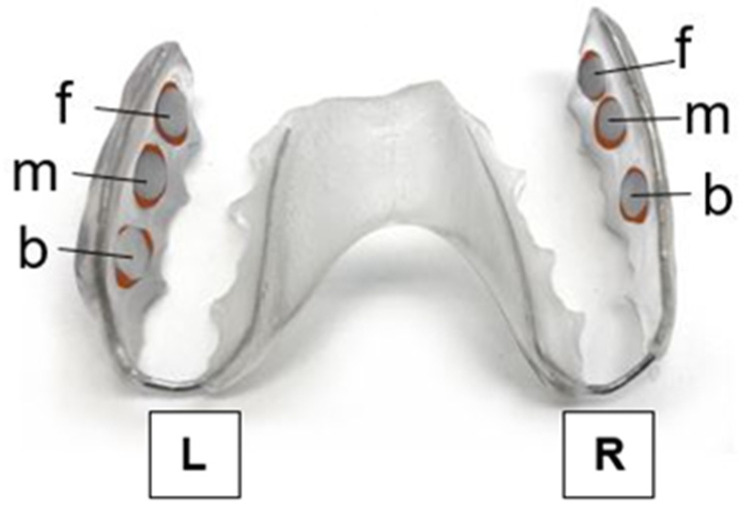
Individual upper-jaw acrylic appliance with the enamel slabs placed in different locations on each side in front (f), in the middle (m), or at the back (b). The samples were positioned on the right (R) and left (L) in the splint. The exposed surfaces were attached to the splint with adhesive wax.

Individual upper-jaw acrylic splints were fabricated for each study participant. The plastic splints were disinfected with 70% ethanol before insertion. The enamel platelets were fixed in the depressions with adhesive wax (Supradent; Oppermann-Schwedler, Bonn, Germany) immediately prior to the start of the timed period, whereby the enamel surface was not covered by adhesive wax and remained untouched. The surfaces of the enamel platelets were at a distance of approximately 1 mm from the buccal surfaces of the posterior teeth. In this position, normal saliva flow and protection against manipulation from the tongue or cheek could be ensured (Figure 1).

### 2.2. Extract Preparation

The *I. viscosa* extract was prepared using pressure liquid extraction. A Dionex 300 system (ASE, accelerated solvent extraction) with 100 mL stainless-steel vessels was used. In the following, 20 g of ground *I. viscosa* aerial parts was introduced into the cells. Extraction was carried out under the following conditions: 70 °C, 120 bar, 1 min preheating time, 5 min heating time, two extraction cycles of 5 min each, 100% flush volume, and 120 s cleaning. The final ethyl acetate extract was then produced using a rotary evaporator until it was dried at 40 °C under reduced pressure. The yield was 2.08 g of ethyl acetate extract [19].

### 2.3. Protocol for Treatment of the Initial Adhesion

Each specimen covered with initial biofilm was assigned a later treatment, with the controls on each side (negative control: 0.9% NaCl, positive control: 0.2% chlorhexidine (CHX), toxicity control: 10% dimethyl sulfoxide (DMSO)), and on the opposite side, the treatments with the extract to be tested. Each volunteer carried an individual upper-jaw acrylic appliance to which six specimens were fixed for two hours. This procedure was performed twice for each subject. After the initial adhesion had been obtained in situ, the appliances were removed from the oral cavity. Sterile tweezers were used to detach the adhesive wax from the samples, which were then rinsed off with sterile 0.9% NaCl for 30 s. The following solutions were prepared in 6 wells in a 24-well plate (µ-Slide 8 Well; Ibidi GmbH, Munich, Germany): 0.9% NaCl, 0.2% CHX, 10% DMSO. The extract to be tested was vortexed in 300 µL of DMSO until it was completely dissolved. This was followed by dilution with phosphate-buffered saline (PBS) so that the corresponding concentrations of the natural substances to be tested were reached. The adherent bacteria on the enamel platelets were treated in the solutions for 10 min.

### 2.4. Determination of Colony-Forming Units (CFU)

For the quantification of viable bacterial counts, the specimens with the adherent bacteria were transferred to Eppendorf tubes after the 10 min treatments with the *I. viscosa* extract. Each Eppendorf tube contained 500 µL of 0.9% NaCl solution. To desorb the microorganisms, the samples were placed in an ultrasonic bath (Sonorex Digital 10p; Bandelin, Berlin, Germany) for 1 min. The Eppendorf tubes were then carefully vortexed, and the specimens were removed with sterilized dental tweezers. A dilution series was prepared to obtain countable single bacterial colonies. This was carried out by diluting them to 1:5, 1:50, and 1:500 with 0.9% NaCl solution. After further vortexing, 100 µL of the solutions that remained was removed from each tube with a pipette and plated on agar plates. Yeast-cysteine blood agar (HCB) plates were used to cultivate anaerobic bacteria at 7 °C, and Columbia blood agar (CBA) plates were used to enable the growth of aerobic and facultative anaerobic bacteria at 5–10% CO_2_ and 37 °C. The CBA plates were incubated for 5 days, whereas the HCB plates were incubated for 10 days using GasPaks (GENbox^®^ Anaer GasPaks; bioMérieux, Marcy l’Etoile, France). At the end of the incubation period, the CFUs were counted for each plate and a CFU mean value was calculated from the identical dilutions and incubation conditions. The number determined was based on the intraorally exposed surface of the samples of 0.196 cm^2^ (π × 2.5^2^ cm^2^), taking the respective dilution into account, to calculate the number of CFU per cm^2^.

### 2.5. Live/Dead Staining and Fluorescence Microscopy

For the live/dead staining and fluorescence microscopy (FM) assay, the fluorescent SYTO 9 stain and propidium iodide (PI) (Live/Dead BacLight bacterial viability kit; Life Technologies GmbH, Darmstadt, Germany) were used. The two dyes, SYTO 9 and propidium iodide (PI), were mixed 1:1 and added to the samples [35]. The green fluorescent nucleic acid SYTO 9 penetrates living and dead bacterial cells. When SYTO 9 binds to nucleic acids, the fluorescence signal is amplified, in contrast to unbound SYTO 9. In order to be able to differentiate dead cells, the second dye (PI) is required [36]. This dye only penetrates cells whose membrane is damaged, and there binds to nucleic acids, which appear red under a fluorescence microscope. Components A (SYTO 9, 1.67 mM) and B (PI, 1.67 mM) were mixed 1:1. This mixture was then diluted with 0.9% NaCl solution to achieve a final concentration of 0.1 pM. The enamel samples were subsequently stained in the solution with the intraorally exposed side facing upward at room temperature and in a dark room for 10 min. Any residues were then washed off by swirling the platelets several times in 0.9% saline solution. Afterwards, the specimens were placed with the plaque side down on a drop of 0.9% NaCl solution in an 8-chamber cover disk (μ-Slide 8 Well; ibidi, Munich, Germany) and were analyzed using FM with a 63× oil immersion objective (ApoTome.2, Zeiss, Oberkochen, Germany). For quantification, 10 representative locations on the enamel sample surface were selected, which resulted in 60 images to be evaluated per test person. The respective living or dead bacteria on these were then determined using the image analysis program ZEN 2 pro (Zeiss, Oberkochen, Germany). The data obtained were used to calculate the coverage rates from living and dead bacteria [37]. Representative images were acquired for demonstration of the results.

### 2.6. Statistical Analysis

For the descriptive analysis of the data, the mean values and standard deviations were calculated and a graphic representation with boxplots was carried out. Diagrams of the viable bacterial counts on the log_10_ scale per cm^2^ (log_10_/cm^2^) were graphically displayed. Linear mixed models with the patient as the random effect were used to analyze treatment differences in bacterial counts, and the Bonferroni method was used to correct for multiple testing. All calculations were carried out with STATA (StataCorp LT, College Station, TX, USA, version 14.1) and the level of significance was *p* < 0.05.

## 3. Results

### 3.1. I. viscosa Extract Significantly Decreased the Viable Counts of Oral Microorganisms during Initial Adhesion

**Figure 2:**
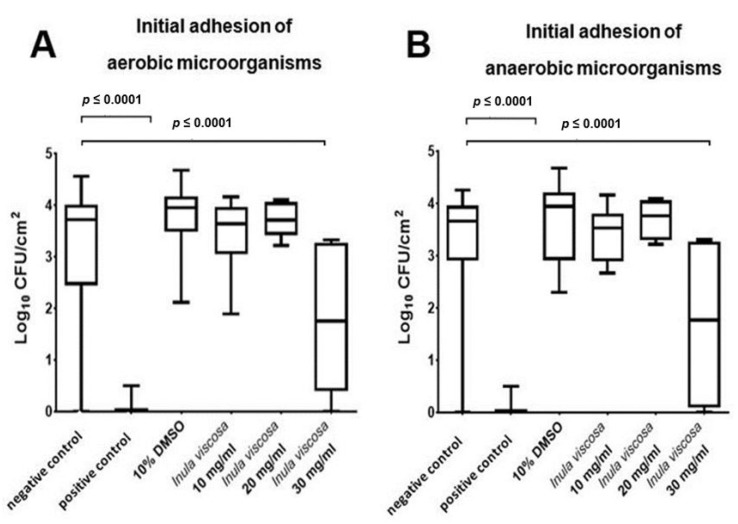
The graphs show the number of CFUs that demonstrate the antimicrobial effect of the tested substances on aerobic (**A**) and anaerobic (**B**) bacteria after an oral exposure time of two hours. An untreated negative control (NaCl 0.9%), a positive control (CHX 0.2%), and a control with DMSO (10%) were also used, as was the natural *I. viscosa* extract (10, 20, and 30 mg/mL) with a 10 min exposure time. The CFU values were shown on a log_10_ scale per cm^2^ (log_10_/cm^2^). The box shows the area in which the middle 50% of the data lies. The line dividing the box shows the median. The *p*-values of the significantly different data are marked on the graphs.

Figure 2A,B show the high eradication rates of initially adherent oral aerobic (Figure 2A) and anaerobic (Figure 2B) microorganisms after the treatment with *I. viscosa* extract at a concentration of 30 mg/mL, plus the untreated negative (NaCl) and positive (CHX) controls. The *I. viscosa* extract induced a substantial reduction of more than 99.99% in the viable bacterial count after two hours of initial microbial adhesion in situ. When using *I. viscosa* at a concentration of 30 mg/mL, a log_10_ CFU value of 1.837 ± 1.54 (median 1.754) was found. Thus, there was a highly significant reduction (*p* < 0.0001) in CFU compared with the negative control and with the enamel platelets treated with DMSO. With regard to the adherent aerobic oral microorganisms (Figure 2A), the untreated control showed a log_10_ CFU value of 3.299 ± 1.045 (median 3.772). The positive control showed a significant reduction in viable bacteria of more than 99.99% (log_10_ CFU value of 0 ± 0) after 10 min of treatment with 0.2% CHX. The treatment with DMSO (10%) did not result in any significant change of viable bacterial count compared to the negative control (log_10_ CFU value 3.796 ± 0.6412; median 3.946). No killing effects were determined after treatment with *I. viscosa* at concentrations of 10 mg/mL (log_10_ CFU value 3.776 ± 0.8306; median 3.776) and 20 mg/mL (log_10_ CFU value of 3.725 ± 0.2999; median 3.711). Figure 2B shows the log_10_ counts of adherent anaerobic bacteria on the enamel samples after 10 min of treatment with NaCl, CHX, DMSO, and *I. viscosa* extract (10, 20, and 30 mg/mL). The negative control had a CFU value of 3.226 ± 1.17 log_10_ (median 3.662). Here, a significant reduction (*p* < 0.0001) of adherent microorganisms during treatment with the positive control (log_10_ CFU value 0.0383 ± 0.1382; median 0) was also demonstrated.

The toxicity control with DMSO (log_10_ CFU value 3.7 ± 0.8072; median 3.944) showed no significant change (*p* ≥ 0.05) compared to the negative control. Treatment with *I. viscosa* extract at concentrations of 10 mg/mL (log_10_ CFU value 3.425 ± 0.534; median 3.532) and 20 mg/mL (log_10_ CFU value 3.706 ± 0.4028; median 3.758) did not yield a significant reduction. However, the anaerobic CFUs decreased significantly (*p* < 0.0001) after treatment with 30 mg/mL of *I. viscosa* extract in comparison to the treatments with NaCl and DMSO. The result was a log_10_ CFU value of 1.71 ± 1.774 (median 1.768).

### 3.2. Live/Dead Assays Revealed High Bactericidal Activity for I. viscosa Extract against Oral Initial Adhesion

**Figure 3:**
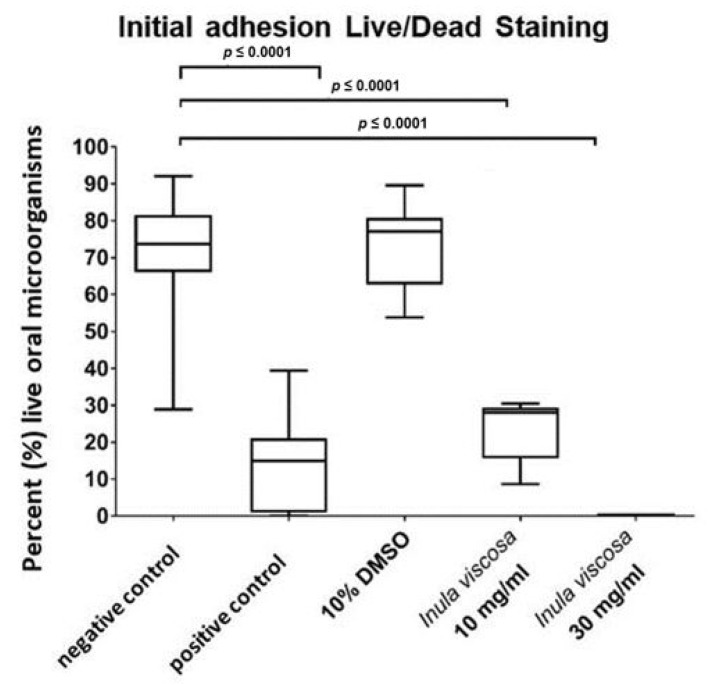
The boxplots represent the percentage of vital oral microorganisms that were evaluated by live/dead staining under the fluorescence microscope (FM). A negative control (NaCl 0.9%), a positive control (CHX 0.2%), a toxicity control (DMSO 10%), and the initial biofilm treated with *I. viscosa* extract at different concentrations (10 and 30 mg/mL) were evaluated. The line dividing the box shows the median. The *p*-values of the significantly different data are marked on the graphs.

The quantitative results of the remaining vital bacteria detected by the live/dead assay during initial adhesion two hours after treatment with *I. viscosa* extract in two concentrations (10 and 30 mg/mL) are depicted in Figure 3 in the form of boxplots. In the negative control (Figure 3), 68.93% of the bacteria adhering to the enamel were vital (±26.89, median 75.0). In the positive control (treatment with 0.2% CHX), the coverage rate of vital bacteria was significantly reduced (*p* < 0.0001) to 16.59% (±29.05 median 15.8). The toxicity control showed 69.99% vital adherent microorganisms (±21.3, median 77.52). After treatment with *I. viscosa* extract at a concentration of 10 mg/mL, there was a significant reduction (*p* < 0.0001) compared to the negative and toxicity control, with only 20.87% of the bacteria being vital (±22.86, median 27.4). In an *I. viscosa* extract concentration of 30 mg/mL, no vital microorganisms could be detected.

**Figure 4:**
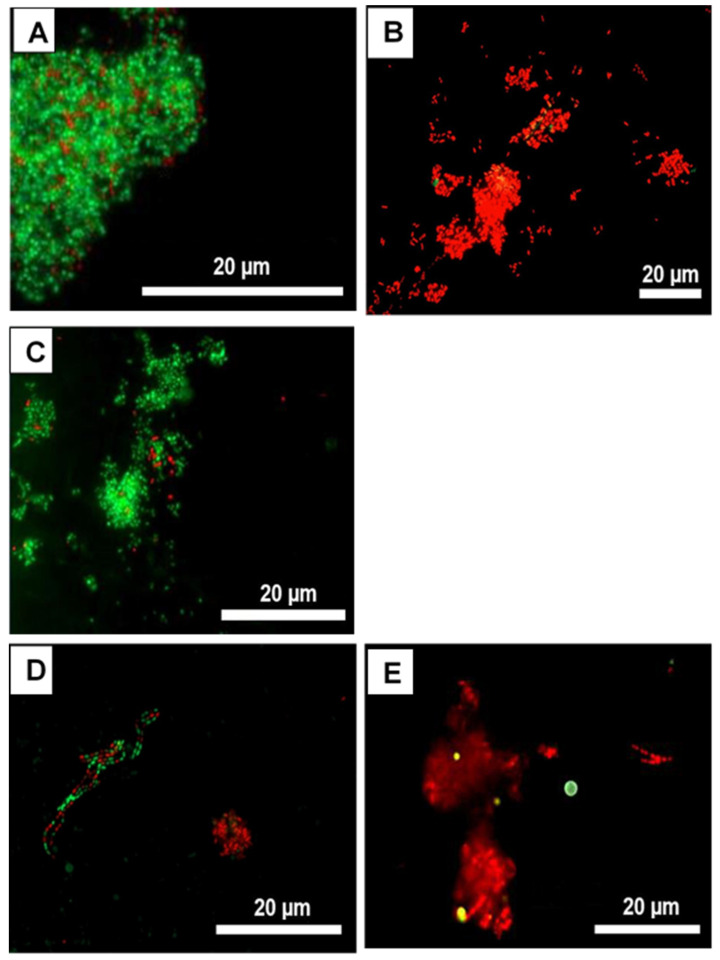
Fluorescence microscopy (FM) images after live/dead staining with BacLight^®^. The vital bacteria fluoresce in green, the avital in red. The effects on the initial adhesion (2 h) after 10 min of 0.9% NaCl treatment (negative control) (**A**), 0.2% CHX treatment (positive control) (**B**), 10% DMSO treatment (toxicity control) (**C**), as well as after treatment with *I. viscosa* extract in two concentrations: 10 mg/mL (**D**) and 30 mg/mL (**E**), are shown.

Figure 4 shows representative FM images of live/dead-stained initial oral biofilms two hours after the treatment with different concentrations of the *I. viscosa* extract. In the untreated control (Figure 4A) and the control treated with DMSO (Figure 4C), a dense accumulation of viable (green) bacteria was detected on the specimens. Very few cells were avital (red). Most microorganisms exhibited diverse arrangements of single cocci, mono- or multi-stratified chains, and three-dimensional bacterial aggregates varying in size. In contrast to the negative controls, the structure of initial oral biofilms treated with CHX (Figure 4B) and *I. viscosa* extract (Figure 4D,E) was markedly different. In fact, most of the initially adhered bacteria were avital (red), which aligns with the results shown in Figure 3.

## 4. Discussion

For the first time, the present study established *I. viscosa* extract as a potent agent with an antimicrobial effect on initial oral biofilms in situ.

Enamel samples from BSE-negative cattle were used, as constant quality is assumed for bovine front teeth [38,39]. In addition, bovine enamel is very similar to human enamel in terms of its physico-chemical structure, mineral composition, density, and structure [40,41]. Bovine enamel is also the most similar to human enamel when comparing the enamel of sheep, cattle, and pigs [42]. After successful production, the enamel platelets were treated with various reagents (sodium hypochlorite, ethanol, double-distilled water) in an ultrasonic bath in order to rid the enamel samples of bacteria [43]. The enamel samples were stored in distilled water until they were used, so that a hydration layer could be formed on the clean enamel surface. This is a prerequisite for the in situ pellicle formation without the effect of surface-active substances [44]. The approach used in the present work to obtain an initial biofilm by means of bovine enamel platelets attached to a plastic splint has frequently been used and is described in previous clinical studies [45,46]. The CFU method was used to determine the total adherent number of bacteria on the enamel slabs. Individual or aggregated bacterial cells from a single colony were cultivated. The CFU quantification showed high standard deviations, which can be attributed to the fact that active bacterial communities are heterogeneous, dynamic systems that are influenced by multiple internal and external factors, and in which death and growth processes constantly occur in the framework of homeostasis [47]. Individual differences in the microbial counts among the samples can also lead to high standard deviations. Furthermore, extremely high CFU numbers can lead to errors in quantification, due to overlay or accumulation [48]. The large dispersion could be explained by the fact that biofilm formation is also a dynamic process and includes various cell–cell communications, such as quorum sensing and horizontal gene transfer. The complexity of a structured microbial biofilm affords microorganisms a high resistance against diverse antimicrobials and distinct physical and chemical properties, which enhance the biofilm resilience [3]. The use of a live/dead viability assay not only supplemented the CFU quantification but also enabled visualization of the *I. viscosa*-treated initial oral biofilms. Vital staining with the BacLight^®^ kit using the dyes SYTO 9 and propidium iodide (PI) is a reliable method to differentiate vital from non-vital microorganisms [49]. The examination took place under a fluorescence microscope (FM). In comparison to conventional methods such as light microscopy, FM achieves high optical resolutions. FM is ideal for examining cell physiology, as it is specified for proteins, lipids, or ions in cells [50]. When counting under the FM, 10 different representative grid fields were selected for each melt platelet. Different bacterial agglomerates could be recorded and counted. This confirmed a high intra- and inter-individual variability in the distribution of live/dead bacteria within the biofilm as well as the resulting coverage rates, as already shown in earlier studies [49,51]. The determined coverage rates are therefore just an approximation and only serve as a basic assessment of the relationship between vital and non-vital microorganisms. A major limitation of FM was the fact that natural extracts, namely *I. viscosa*, showed a high level of self-fluorescence. Thus, some areas on the enamel slabs could not be evaluated because of the high levels of self-fluorescence of the extract. However, there were still enough spots to allow for a proper quantification. The total number of vital microorganisms declined significantly after the treatment with *I. viscosa* extract. In the present work, the differences between the vital staining and the CFU can be attributed to the fact that bacterial aggregates are shaken up by the ultrasonic bath and thus falsify the result, as without treatment they might only have formed a single CFU [52].

In a previous report, Karygianni et al. tested the effect of both the ethyl acetate extract and the methanol extract of *I. viscosa* on certain planktonic oral bacterial species [14]. The microbial species were *S. mutans*, *S. sobrinus*, *S. oralis*, *Enterococcus faecalis*, *C. albicans*, *Escherichia coli*, *S. aureus*, *P. gingivalis*, *P. intermedia*, *F. nucleatum,* and *Parvimonas micra*. This study revealed that the minimum inhibitory concentration (MIC) of *P. gingivalis* was 0.07 mg/mL, and *S. sobrinus*, *E. faecalis,* and *E. coli* showed an MIC of up to 2.50 mg/mL. At a minimum bactericidal concentration (MBC) of 0.15 mg/mL, obligate anaerobes such as *P. gingivalis* could be eliminated, whereas *E. faecalis* was more resistant. Taking the above results into account, one could have expected a significant reduction of the initial adherent bacteria at a concentration of 10 mg/mL. However, the concentration had to be increased up to 30 mg/mL to observe a significant effect on the number of CFUs. Our previously reported results on the effects of *I. viscosa* extract on diverse planktonic bacteria provide a strong indication of possible bacteriostatic effects on the initial oral adhesion. The antibacterial effect can mainly be attributed to the polyphenols and the essential oils contained in *I. viscosa*. These oils are flavonoids/aglycones, genkwanin, naringenin, quercetin, camphor, luteolin, and apigenin, whose antimicrobial effects have been proven [53,54,55]. Particularly apigenin, luteolin, and naringenin show high antibacterial effectiveness against oral streptococci, which make up the largest portion of oral microflora [56,57,58]. Regarding the antimicrobial effects of the ingredient apigenin, it was shown that it inhibits glycosyl-transferases [57], resulting in the production of fewer glucans required for the synthesis of extracellular polymeric substances (EPS), a compound crucial for biofilm formation and microbial resistance [59]. Quercetin has been shown to be able to damage the cell walls of *Bacillus cereus* and *C*. *albicans* [25]. Furthermore, polyphenols also have an antimicrobial effect on the growth of biofilms by denaturing or obscuring functional groups of the receptor proteins and thus reducing the interaction between different bacterial species. Therefore, a negative effect on biofilm formation could also be achieved with *Cistus* tea, which is rich in polyphenols [60]. Hertel et al. investigated the effect of *I. viscosa* tea on initial oral biofilm formation [27]. The test subjects carried splints with bovine enamel slabs similar to the ones used in the present study and rinsed for 10 min with *I. viscosa* tea, and afterwards the splints remained intraorally for eight hours. It could be shown that the percentage of viable adherent bacteria was affected by the tea. After rinsing with *I. viscosa* tea, the proportion of dead bacteria increased (38% viable: 62% dead). In the present study, the inhibitory effect was even stronger, as *I. viscosa* extract at a concentration of 10 mg/mL led to 21% viable and 79% dead bacteria, whereas 30 mg/mL yielded even better results (0% viable: 100% dead). This discrepancy in outcomes could be attributed to the fact that the boiled *I. viscosa* tea reduced the concentration of the actual effective ingredients to a concentration lower than 10 or 30 mg/mL. The difference between the two studies was that Hertel et al. [27] examined bacterial growth after rinsing with *I. viscosa*, whereas the current study investigated how the extract affected the microbiological composition after bacterial colonization. Our data do not support that an *I. viscosa* extract (10 or 20 mg/mL) can replace common mouthwash solutions such as CHX (0.2%) since the effect is inferior to that produced by 0.2% CHX, which almost always resulted in no viable bacterial species. However, *I. viscosa* extract at a concentration of 30 mg/mL is certainly a bacteriostatic alternative, as the CFU could be significantly reduced at this concentration. A comprehensive testing of a dilution series that includes both lower concentrations and concentrations higher than 30 mg/mL is necessary to identify the optimal inhibitory concentration of the extract and to evaluate a possible higher eradication effect on the initial and mature oral biofilm. Moreover, the reported adverse effects of frequent use of CHX solution in the oral cavity should be taken into consideration in the comparison to mouthwashes based on natural products [17,18]. Even if it seems generally accepted that natural products are safe and devoid of side effects, natural products can also have toxic properties, react with other drugs, and cause severe side effects [61]. They can also cause allergies, for example contact dermatitis after the use of *Magnolia officinalis* and *Allium sativum* [62]. *Verbena officinalis* can also cause gastrointestinal dysfunction and heartburn [63,64]. Therefore, interactions with other drugs should always be monitored and adequately tested. The identification of single active components of the tested extract and an analysis of their antimicrobial activity should also be conducted in future studies since the concentration of such ingredients can depend on the geographical location the plant was harvested in. Additionally, there are some indications in the literature that *I. viscosa* extract inhibits cancer cells at concentrations of 150 or 300 mg/kg in mice experiments [65]. Hence, the toxicity of the *I. viscosa* extract used towards human oral mucosal cells should be examined in future studies. In a comprehensive phythochemical analysis, Mahmoudi et al. [66] reported that *I. viscosa* leaves are rich in the unsaturated essential fatty acids (UFAs) α-linolenic (C18:3) and linoleic (C18:2) acid. The authors recommended the use of *I. viscosa* as a safe source of such essential UFAs and hence as a beneficial supplement to the human diet. Furthermore, in the total leaf lipid fraction of *I. viscosa*, the authors found a ω-3/ω-6 ratio of 4.42, which is similar to the intake values (5) recommended by nutritionists. Additionally, a high total phenol content (TPH) and total flavonoid content (TFC) were determined in *I. viscosa* leaves [66]. Considering the reduction of both azino-bis(ethylbenzothiazoline 6-sulfonic acid) and 2, 2-diphenyl-1-picrylhydrazyl, the high antioxidant effect of these contents that was shown by the authors again indicates a possible use of certain compounds from the *I. viscosa* extract as a food supplement [66]. In general, the phenolic compounds identified in *I. viscosa* comprised caffeic acid-O-hexoside, p-coumaric acid, chlorogenic acid, 1,3-O-dicaffeoylquinic acid, taxifolin hexoside, hydroxybenzoic acid hexoside, isorhamnetin-O-hexoside, 3,4-dicaffeoylquinic acid, 3,5-dicaffeoylquinic acid, 4,5-dicaffeoylquinic acid, coumaroyl caffeoylquinic acid, dimers of caffeic acid-O-hexoside, luteolin, isorhamnetin-3-O-(6-O-feruloyl)-glucoside, isorhamnetin, acetyl taxifolin, and an unknown dihydroflavonol [66]. Many of these compounds contribute to the antioxidant and antifungal activity reported for *I. viscosa* extract [29]. Considering the aforementioned detailed composition of *I. viscosa* leaves, *I. viscosa* could be used as a source of bioactive components such as phenolic compounds and volatile oils. Additionally, its antioxidant, antibacterial, and antifungal properties indicate its potential use for the development of natural preservatives with applications in agro-food.

## 5. Conclusions

In conclusion, the growing relevance and importance of phytotherapy for providing novel treatments in the field of dental research can be clearly shown. Many natural extracts have the potential to feature extensive medicinal efficacy and could therefore supplement, or even completely replace, chemically produced drugs in the long term. However, further clinical studies must be carried out to be able to comprehensively demonstrate the interactions of the natural extracts within the human body and with other pharmaceuticals. Additionally, clinical studies are required to evaluate such a mouthwash product and to compare it with standard mouth rinses based on CHX or cetylpyridinium chloride (CPC). On the other hand, the properties of the natural extracts such as their consistency, solubility, and interactions with surfaces must be investigated in order to be able to guarantee a user-friendly, durable, and stable application as a common mouthwash solution. The antioxidant, antibacterial, and antifungal activities of *I. viscosa* extract indicate its potential use for the development of natural preservatives with applications in agro-food.

## Data Availability

The data are available upon request from the authors.

## Supplementary Materials

The following are available online at https://www.mdpi.com/article/10.3390/nu13114029/s1, Table S1: Overview of the reagents used, including their brands and the countries where they were purchased.
