# Supplementary material for: Antimicrobial Effects of Inula viscosa Extract on the In Situ Initial Oral Biofilm
Source: Nutrients. 2021 Nov 11;13(11):4029. doi: 10.3390/nu13114029 (PMC8622444; doi:10.3390/nu13114029)
Supplement: Supplementary file 1 [file nutrients-13-04029-s001.zip › nutrients-1393311-supplementary.pdf]

nutrients

MDPI

Article

Antimicrobial Effects of *Inula viscosa* Extract on the In Situ Initial Oral Biofilm

Hannah Kurz [1], Lamprini Karygianni [2], Aikaterini Argyropoulou [3], Elmar Hellwig [1], Alexios Leandros Skaltsounis [3], Annette Wittmer [4], Kirstin Vach [5] and Ali Al-Ahmad [1,*]

[1] Department of Operative Dentistry and Periodontology, Medical Center, University of Freiburg, Faculty of Medicine, University of Freiburg, Freiburg, Germany; hannahkurz@gmx.net; elmar.hellwig@uniklinik-freiburg.de, ali.al-ahmad@uniklinik-freiburg.de
[2] Clinic of Conservative and Preventive Dentistry, Center of Dental Medicine University of Zurich, Switzerland; lamprini.karygianni@zzm.uzh.ch
[3] Department of Pharmacognosy and Natural Products Chemistry, Faculty of Pharmacy, National and Kapodistrian University of Athens, Athens, Greece
[4] Institute of Medical Microbiology and Hygiene, Faculty of Medicine, University of Freiburg, Freiburg, Germany
[5] Institute for Medical Biometry and Statistics, Faculty of Medicine and Medical Center, University of Freiburg, Freiburg, Germany
* Correspondence: ali.al-ahmad@uniklinik-freiburg.de; phone: +49 761 27048940

Table of Contents:

Table S1: Overview of the reagents used, including their brands and the countries where they were purchased

Table S1: Overview of the reagents used, including their brands and the countries where they were purchased

**Reagent**	**Brand**	**Country**
Aqua ad injectabilia	B. Braun	Melsungen, Germany
Chlorhexidine	Pharmacy, University of Freiburg, Faculty of Medicine	Freiburg, Germany
Aqua destillata	Pharmacy, University of Freiburg, Faculty of Medicine	Freiburg, Germany
Dimethyl sulfoxide	Merck	Hohenbronn, Germany
Ethanol	Honeywell	Seelze, Germany
Formic Acid	Honeywell Morristown	New Jersey, USA
Live/Dead BacLight bacterial viability kit: 1.67 mM SYTO 9 nucleic acid stain, 1.67 mM propidium iodide	Life Technologies GmbH	Darmstadt, Germany
Sodium chloride (0.9)%	B. Braun	Melsungen, Germany
Sodium hypochloride	Hedinger GmbH	Stuttgart, Germany
Phosphate-buffered saline	Institute of Medical Microbiology and Hygiene, Faculty of Medicine, University of Freiburg	Freiburg, Germany
Thymol (99%)	Carl Roth	Karlsruhe, Germany
